# Anal incontinence and unrecognized anal sphincter injuries after vaginal delivery– a cross-sectional study in Norway

**DOI:** 10.1186/s12905-020-00989-5

**Published:** 2020-06-22

**Authors:** Matilde Risopatron Berg, Ylva Sahlin

**Affiliations:** 1 Department of Colorectal Surgery, Innlandet Hospital Trust Hamar, Hamar, Norway; 2 University of Oslo, Faculty of Medicine, Institute of Clinical Medicine, Oslo, Norway; 3 Innlandet Hospital Trust Hamar, Hamar, Norway

**Keywords:** Anal incontinence, Obstetric anal sphincter injury, Obstetric care, Occult injury

## Abstract

**Background:**

Aim of the study was to estimate the prevalence of postpartum anal incontinence among women who delivered vaginally, and to assess the extent to which obstetric injuries to the anal sphincters are missed.

**Methods:**

All women (both primiparous and multiparous) who delivered vaginally and received any kind of sutures in the perineal area at Innlandet Hospital Trust Elverum in Norway between January 1, 2015 and June 30, 2016 were invited to answer a questionnaire on St. Mark’s incontinence score and to participate in a clinical examination of the pelvic floor including endoanal sonography.

**Results:**

In total 52,3% (*n* = 207) of the 396 invited women participated in the study. Mean St. Mark’s score was 1.8 points (95% CI 1.4 to 2.1) at examination 14 months (mean) postpartum, and none of the participants suffered from weekly fecal leakage. Fecal urgency affected 11.7% (95% CI 7.1 to 16.3) of the participants, and 8.7% (95%CI 5.1 to 12.8) had weekly involuntary leakage of flatus. Nine women (9.3%, 95% CI 4.1 to 15.5) had a previously undetected third degree obstetric anal sphincter injury.

**Conclusion:**

The prevalence of anal incontinence among women who have delivered vaginally and received sutures due to 1st and 2nd degree perineal lacerations is low. Some obstetric anal sphincter injuries remain unrecognized at the time of delivery, but the symptoms of anal incontinence due to these injuries are in the lower half of the St. Mark’s incontinence score. Women with persistent symptoms like fecal urgency or leakage of gas and/or feces should be referred to evaluation by a colorectal surgeon in order to achieve optimal treatment.

## Background

Vaginal delivery is known to be one of the risk factors for anal incontinence among adults, and women with obstetric anal sphincter injury (OASIS) have higher risk of severe anal incontinence than women without such injury [1, 2]. The prevalence of anal incontinence among women who sustained OASIS 1–5 years previously has been found to be 18–53% for flatus and 3–23% for stools [3–7]. Among parous women without OASIS, the prevalence of incontinence to flatus was 15–32% and incontinence to stools 5–7% at 4–5 years postpartum [3, 4].

Studies show a prevalence of 0,5–5% recognized obstetric anal sphincter injuries (OASIS) [5, 8, 9]. In Norway, the prevalence of recognized OASIS has decreased in recent years [8] and was diagnosed in 1.8% of all vaginal deliveries between 2015 and 2016 according to the Medical Birth Registry in Norway [10]. The decrease in OASIS happened after implementation of a national intervention program for improved delivery techniques for protection of the perineum in Norway [8].

When a third or fourth degree injury is recognized immediately after delivery, reconstructive surgery is performed. However, more than half of OASIS are not recognized in the delivery ward [11–13]. Andrews et al. showed that most unrecognized OASIS are detectable by re-examination by trained examiners, the prevalence of OASIS in their study of 254 vaginal deliveries increased from 11% to 24,5% when women were re-examined. Only 1,2% of the OASIS detected in their material were truly occult injuries [11]. In another study by Groom et al. increased vigilance improved the diagnosis of OASIS from 2,5% recognized injuries prior to the intervention study, to 9,3% during the intervention period [12].

This study includes both primiparous and multiparous women who delivered vaginally no earlier than 4 months before study participation. This was necessary in order to assess whether or not symptoms of anal incontinence persisted postpartum, and to achieve reliable conclusions from the endoanal ultrasonography. The aim of this cross-sectional study was to evaluate the degree to which anal incontinence affects women who have not sustained OASIS during vaginal delivery. We also wanted to investigate whether there are unrecognized OASIS despite the improvement in preventive delivery techniques in Norway in recent years. This study adds value to the literature in that the prevalence of anal incontinence and unrecognized OASIS were evaluated several months postpartum, during which the tissues of the pelvic floor have had time to heal.

## Methods

Participants were recruited among women who gave birth vaginally and received any kind of sutures in the genital area afterwards at Innlandet Hospital Trust in Elverum, Norway, during the period January 1, 2015 to June 30, 2016. Both primiparous and multiparous women were invited to study participation. It was assumed that those patients who received sutures were more likely to be among those who had at least some degree of perineal tear as compared to those who did not receive sutures. Women with recognized third and fourth degree OASIS were excluded. Women who had delivered vaginally again less than 4 months prior to study participation were also excluded.

Invitations to study participation and questionnaires were sent by mail to women given the procedure code for sutures after vaginal delivery in the period January 1, 2015 to June 30, 2016. Reminders were sent to non-responders three and six weeks after the first invitation.

All women who accepted to participate gave written, informed consent and filled out a questionnaire, containing demographic variables such as age, parity, height and weight, specific details regarding their deliveries, and the St. Mark’s score to assess symptoms of anal incontinence. The St. Mark’s score was chosen because it includes fecal urgency, and it is a commonly used tool for the assessment of anal incontinence [14]. Furthermore it has been shown to correlate with the patients’ perceptions of their symptoms [15].

Upon clinical examination, the pelvic floor was examined by inspection, palpation, and rectal exploration, during voluntary contraction and relaxation of the pelvic floor. The anal sphincters were assessed by two-dimensional endoanal sonography with motorized crystals (Bk Medical Flex Focus 800). Participants who were pregnant were excluded from participation in the clinical and endoanal sonography examination in order to prevent any unfortunate events such as premature contractions or distress to the fetus.

**Fig. 1:**
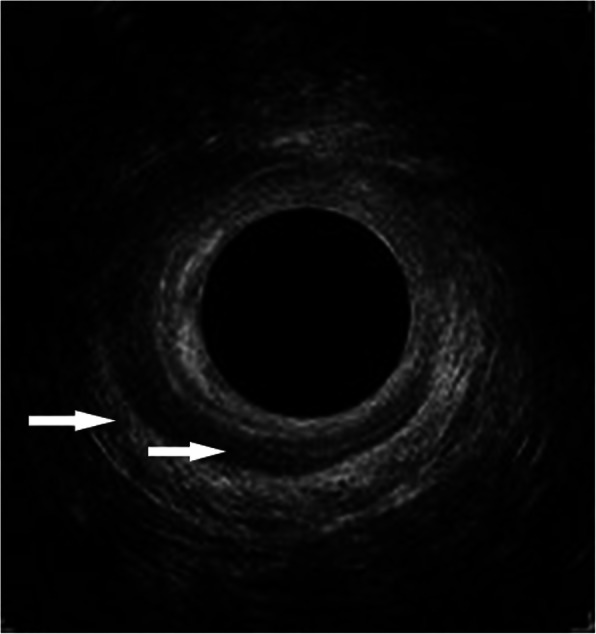
Photograph of endoanal ultrasonography image from the mid anal canal. The hyperechoic outer circle is the external anal sphincter, and a defect is visible from the 9 o’clock position to the 3 o’clock position. The hypoechoic inner circle is the internal anal sphincter, and a defect is visible from the 11 o’clock position to the 4 o’clock position. Arrows point to each of the sphincters

Defects in the anal sphincters were defined as a visual gap in each of the anal sphincters equivalent to at least four clock-hours and encompassing at least 50% of the height from proximal to distal in the sagittal plane of the anal sphincter in question (Fig. 1). Complete ruptures were defined as injuries affecting the complete height and thickness of the anal sphincter, whereas partial ruptures were defined as injuries affecting at least half the height of the anal sphincter, but less than the complete height.

Two researchers, one of which who has many years of experience with surgical treatment of OASIS, performed all clinical examinations together, including the endoanal sonography. The distance from the anus to the posterior commissure of the vagina was measured in centimeters. The height of the anal sphincter complex was measured anteriorly at the twelve o’clock position in centimeters as the ultrasound probe moved from the lower border of the internal sphincter up to the point where the puborectal muscle appeared.

The participants were grouped according to participation or not in the clinical examination (participation or not participation), and those who took part in the clinical examination were also grouped by the status of the anal sphincter complex (intact or injured).

The statistical analyses were perfomed using SPSS package 24 and Stata SE 15. Means and proportions for the different variables were calculated with 95% confidence intervals and standard deviations. Medians were calculated with interquartile ranges. Participants were grouped according to participation or not in the clinical examination and according to the status of the anal sphincters upon clinical examination. Two- sample independent t-tests were used to compare means between the two groups, and Pearson’s Chi^2^- tests to compare proportions. *P*-values lower than 0.05 were considered significant. Missing data were excluded from analyses.

The study was approved by the Regional Committee for Medical and Health Research Ethics in South and Eastern Norway (case number 344/2016) and by the institutional review board for scientific studies at Innlandet Hospital Trust, which also granted the financial means and facilities to conduct the study.

## Results

**Fig. 2:**
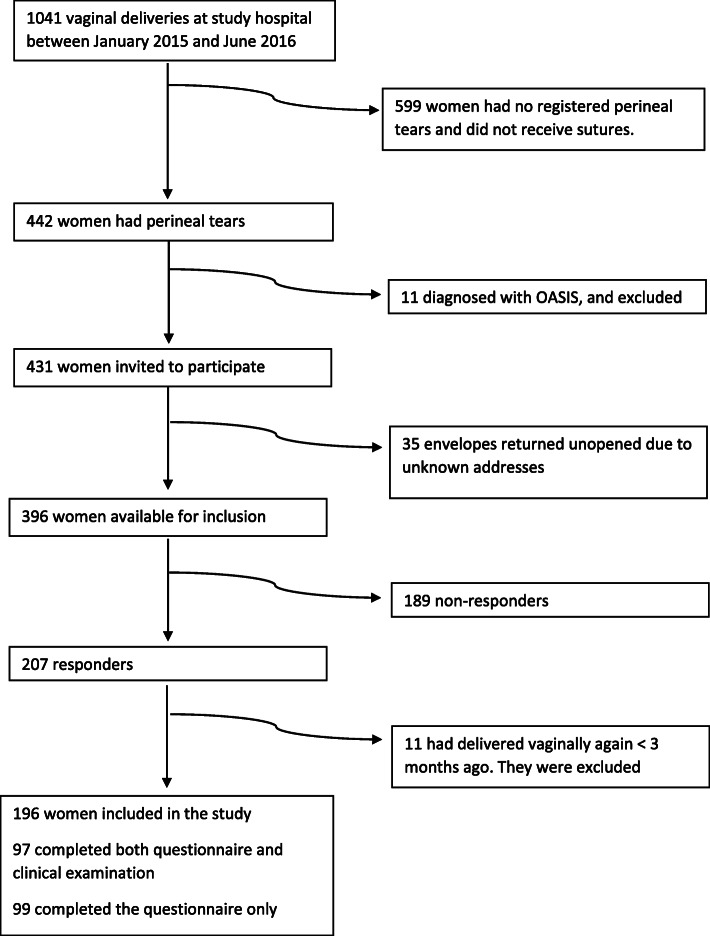
Flowchart of the inclusion of patients

Study participants were recruited between November 2016 and April 2017. A total of 207 women answered the questionnaire (52.3% of the 396 available for inclusion), and 97 of the women also participated in the clinical examination including endoanal sonography by a median time of 15 months postpartum. Eleven women had delivered vaginally again less than 3 months prior to study participation, and were excluded from the analysis. (Fig. 2, Flowchart).

All background variables failed to show significant difference between the women who underwent clinical examination and those who only answered the questionnaire (Table 1).

**Table 1:** Background variables

Back-ground variables	All participants (*N* = 196)		Participants without clinical examination (*N* = 99)		Participants with injury to the anal sphincter (*N* = 9)		Participants with Intact anal sphincter (*N* = 88)	
	**Median**	**IQR**	**Median**	**IQR**	**Median**	**IQR**	**Median**	**IQR**
Age (years)	31	8	31	8	32	11	31	8
BMI	23.9	6.5	23.7	7.2	21.3	9.1	24.2	5.6
vaginal deliveries	1.0	1	2.0	1	1.0	1	1.0	1
Months post-partum	15.0	10	15.0	9	11.0	12	15.0	9
Birth-weight (grams)	3550	680	3533	711	3280	410	3680	663
	**N (%)**	**95% CI**	**N (%)**	**95% CI**	**N (%)**	**95% CI**	**N (%)**	**95% CI**
Instrumen-tal delivery	28 (14.4)	7.2 to 21.6%	14 (13.3)	6.0 to 27.1%	2 (22.2)	0.0 to 44.4%	12 (13.6)	6.8 to 20.5%

Medians and interquartile ranges, divided in groups by examination or not by endoanal sonography, and status of anal sphincters on endoanal sonography

The mean St. Mark’s score was 1.8 points (95% CI 1.4 to 2.1). Weekly episodes of involuntary leakage of flatus were present in 8.7% (95% CI 5.1 to 12.8) of the participants. None had fecal leakage as often as weekly. 11.7% (95% CI 7.1 to 16.3) suffered from fecal urgency. The highest St. Mark’s score in this study was 11.

**Fig. 3:**
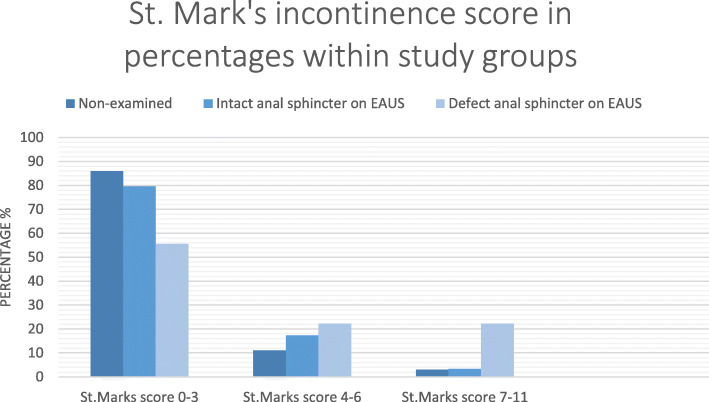
Distribution of St. Mark’s incontinence score among the study participants

Among the 99 participants who only submitted the questionnaire, seven had a St. Mark’s score exceeding five points. Symptoms of anal incontinence had a similar distribution among the participants who only submitted to the questionnaire and the participants found to have intact anal sphincters on the clinical examination. In these groups, only 3.0% (*n* = 3) and 3.4% (*n* = 3) had a St. Marks incontinence score of 7 or higher, versus 22.2% (*n* = 2) of the participants found to have anal sphincter injury on the clinical examination (Fig. 3). Overall, the mean St. Marks incontinence score among the participants who only filled in the questionnaire were significantly lower than among the participants who took part in the clinical examination (*p* = 0,014).

Defects in the anal sphincters were detected in nine of the 97 women who underwent endoanal sonography. The proportion of unrecognized obstetric anal sphincter injuries was 9.3% (95% CI; 4.1 to 15.6%).

Seven out of nine participants with injury to the anal sphincters had partial ruptures, limited to the upper half of the anal sphincters. Complete rupture of the external sphincter was present in two participants, and only one of the participants with partial ruptures had affection of both the external and the internal anal sphincter. Among the nine women found to have anal sphincter injury, three experienced episodes of fecal leakage on a monthly basis, but none as frequent as every week. Two women experienced involuntary leakage of flatus weekly, and four monthly. Three had fecal urgency and one had symptoms limited to dyspareunia. One was asymptomatic.

Instrumental delivery was more common among the women with defects in the anal sphincters than the women with intact anal sphincters, but the difference was not statistically significant (Table 1).

Participants with injury to the anal sphincters had greater St. Mark’s scores than those with intact anal sphincters, 4.7 points versus 2.0 (*p* = 0.007). Shorter perineal body (*p* = 0.019) and reduced height of the anal sphincter complex (*p* = 0.013) characterized the women with defects in the anal sphincters compared to the women with intact anal sphincters (Table 2).

**Table 2:** Clinical variables

	All participants (*N* = 196)		defect anal sphincter (*N* = 9)		Intact anal sphincter (*N* = 88)		Participants without clinical examination (*N* = 99)	
Clinical variables	Mean / %	95% CI	Mean / %	95% CI	Mean/ %	95%CI	Mean/ %	95%CI
St.Marks score	1.8	(1.4–2.1)	4.7*	(2.4–7.0)	2.0 *	(1.5–2.5)	1.3 *	(0.9–1.8)
Weekly Flatus leakage	8.7%	(5.1–12.8)	22.2%*	(0.0–55.6)	12.5%	(5.7–20.5)	4.0%*	(1.0–8.1)
Weekly fecal leakage	0.0%	(0.0)	0.0%	(0.0)	0.0%	(0.0)	0.0%	(0,0)
fecal urgency	11.7%	(7.1–16.3)	33.3%*	(11.1–66.7)	14.8%	(8.0–22.7)	7.1% *	(3.0–12.1)
Length perineal body in CM			2.5 *	**(2.0–3.0)**	3.2*	**(3.0–3.4)**		
Height anal sphincter complex in CM			3.3*	**(2.8–3.8)**	4.1*	**(3.9–4.2)**		

Mean values and proportions with 95%CI, divided in groups by examination or not by endoanal ultrasound, and status of anal sphincters on endoanal ultrasound

*p-value≤0.05 on differences between groups

Among the participants found to have intact anal sphincters, none had experienced fecal leakage weekly or more often, and 12,5% (95% CI 5.7 to 20.5) suffered from involuntary leakage of flatus on a weekly or daily basis. (Table 2).

Age, parity, maternal body mass index and birthweight (of the baby) did not differ significantly between the participants with defects in the anal sphincters and the participants who had intact anal sphincters (Table 1).

Among the participants who underwent clinical examination, we had one missing observation on height of the anal sphincter complex.

## Discussion

The prevalence of anal incontinence in this study was low, and none of the participants had high St. Mark’s scores. Nine women had previously undiagnosed obstetric injury to the anal sphincters. Anal incontinence was more common among the participants with undiagnosed injuries than those with intact anal sphincters, and the women with injuries also had reduced length of the perineal body and height of the anal sphincter complex.

Our findings confirm that not all OASIS are detected in the delivery ward.

Andrews et al. found that 14% of women who deliver vaginally have unrecognized OASIS [11]. Groom et al. performed a study at a hospital in England, and found that the prevalence of OASIS increased from 2,5% before the study to 15% in a group of women who were re-examined by a specially trained research fellow [12]. Our results are a bit lower than the findings of both of these studies from England. The discrepancy could be explained by different delivery techniques and programs for the training of health personell in detecting OASIS at the study hospitals, and in the difference in recruitment of study participants. Especially since our study included both primiparous women and multiparous women, while the other two studies included only primiparous women, which are known to have higher rates of OASIS.

The majority of the women found to have unrecognized injuries in our study had a St. Mark’s score of less than seven, and complete rupture of the external anal sphincter were only present in two women.

Most of the injuries were partial, and the torn part of the anal sphincters were located in the upper half of the muscles. This could complicate an accurate diagnosis in the delivery ward. Our findings imply that it will not be possible to detect all OASIS by the means of rectal exploration and visual inspection of the pelvic floor immediately postpartum. This is consistent with the findings of Frudinger et al. [16], who concluded that sphincter injuries are difficult to detect by clinical examination.

However, at examination by a mean time of 14 months postpartum, we found that the length of the perineal body and the height of the anal sphincter complex upon rectal exploration were significantly shorter in the women with defects in the anal sphincters as compared to those with intact anal sphincters. This is consistent with the findings of Ozyurt et al. [17]. Even though these findings are based on comparision of mean values between women with and without OASIS verified on ultrasonography, obstetricians, gynecologists, general practitioners and physioterapists should be aware that such anatomical findings in parous women could indicate anal sphincter injury and women who experience symptoms should be examined with ultrasonography. Women who develops anal incontinence due to OASIS rarely address this problem when talking to doctors [5, 7, 18], and both gynecologists and other doctors should ask directly about fecal urgency, fecal leakage and involuntary leakage of flatus.

Involuntary leakage of gas was common among the participants found to have intact anal sphincters, but fecal urgency and fecal leakage were more common among the participants found to have unrecognized injuries to the anal sphincters. The participants with unrecognized injuries also had a significantly higher St. Mark’s score. Previous studies have shown that parous women who have sustained OASIS are at greater risk of developing anal incontinence than parous women who have not sustained OASIS. Several case-control studies have been conducted to compare the frequency and severity of anal incontinence among women who have sustained OASIS and women who have not.

A study by Cornelisse et al. in the Netherlands found that 39% of women with OASIS suffered from anal incontinence 4 years postpartum, as compared to 20% of women who delivered vaginally without OASIS [19]. Cornelisse et al. found that most of the women suffered from leakage of flatus, 31% of the women with OASIS and 18% of the women without OASIS experienced this. Soiling were present in 12,1% in the OASIS group and 4,1% in the control group, and leakage of solid stools affected only 1,4% in the OASIS group and 1% of the controls [19].

Similarly, Pollack et al. showed in a Swedish study that 42% of women with OASIS presented with leakage of flatus and 11% with fecal leakage by 5 years postpartum. They also had a control group of women who had delivered vaginally without sustaining OASIS, and found that 27% of controls had leakage of flatus and 5% had fecal leakage [4]. In another Swedish case-control study by Wagenius et al., 33% of women with OASIS experienced leakage of flatus and 21% fecal leakage by 4 years postpartum, versus 15% leakage of flatus and 6% fecal leakage among women who delivered vaginally without sustaining OASIS [3].

In an American study by Evers et al. women with OASIS were compared to women who had delivered vaginally without sustaining OASIS and women who delivered by cesarean section. They found a prevalence of 31% leakage of flatus, 21% fecal leakage among the women with OASIS by five to 10 years postpartum. Among the controls leakage of flatus were present in 23% in the vaginal delivery group and 15% in the cesarean section group, and fecal leakage in 8% in both groups [1].

The same study by Evers et al. found an odds ratio of 2.32 for anal incontinence among women with OASIS as compared to women who delivered by cesarean section. They also showed that the prevalence of anal incontinence and general quality of life were similar between women who delivered vaginally without sustaining OASIS and women who delivered by cesarean section [1].

All of these studies show a significant and strong association between postpartum anal incontinence and sustained OASIS at the index delivery. This is coherent with our findings, that women with OASIS are more likely to develop anal incontinence than women without OASIS.

The study has limitations. The model of recruitment, by which invitations were mailed to women without relationship to the hospital beyond having delivered there, may have contributed to non-response bias. Women who were contacted who did not have symptoms of anal incontinence may not find any personal gain in participating. Non-response bias is plausible, meaning that the true incidence of unrecognized obstetric anal sphincter injuries is probably lower than the 9.3% among our participants.

The main strength of the study is the combination of self-reported symptoms and clinical examination including endoanal sonography, performed at least 4 months postpartum (shortest time between delivery and clinical examination in this study was 4 months, whereas the mean time passed from delivery to examination was 14 months). Four months was set as a cut-off value for inclusion to assure the pelvic floor had had time for natural healing and are no longer swollen due to recent vaginal delivery. Findings of unrecognized OASIS were evaluated together with the symptoms presented by each affected woman. This made it possible to evaluate the degree to which women found to have unrecognized OASIS have symptoms of anal incontinence and are in need of treatment, or not. The endoanal sonography ensures a reliable diagnosis of anal sphincter injuries.

## Conclusions

Our findings indicate that a considerable amount of OASIS is not recognized in the delivery wards. Among women with unrecognized injuries, many will experience anal incontinence. Women with asymptomatic occult anal sphincter injuries are also at increased risk for developing anal incontinence after a subsequent vaginal delivery or later in life [2, 20–22]. Women who presents with persistent symptoms of fecal urgency or leakage of gas and/or feces, could have unrecognized OASIS and should be evaluated by specialist care for treatment. We also suggest that women who deliver vaginally and sustain first or second degree perineal tears should be informed that a small amount of women develop anal incontinence and could have unrecognized injuries to the anal sphincters. They should also be informed to seek medical advice if they experience symptoms of anal incontinence.

## Data Availability

The datasets used and analysed during the current study are available from the corresponding author on reasonable request.

## Abbreviations

OASIS: Obstetric anal sphincter injury
CI: Confidence interval
BMI: Body mass index
cm: Centimeters
